# On Preserving Bodies for Dissection

**Published:** 1845-01

**Authors:** John Inglis Nicol

**Affiliations:** Inverness


*On Preserving Bodies for Dissection.* By John Inglis Nicol, M. D., Inverness.—I read with much interest, in the *Lancet* for the 29th of June last, Mr. Derhust's communication on the subject of Mr. Brookes' method of preserving bodies for dissection, by means of a saturated solution of the nitrate of potash, as it brought before me vivid recollections of a variety of incidents connected with its use.

The colour of the muscles and other textures was preserved while unexposed, for a considerable length of time. In cold weather it answered the purpose admirably; but in the hot season, however, when the parts were once exposed, they speedily lost colour and assumed the usual aspect of decomposition; and this was not all,—under night the subject became infested with maggots, the clearing away of which in the morning was an occupation of the most revolting character, which made the atmosphere of the room peculiarly offensive.* To obviate these annoyances, Mr. Brookes adopted the

*The remarkable rapidity with which these larvæ are produced is well known. I have often seen the cavities of the thorax and abdomen literally filled with them in the morning, although the operations of the previous day rendered scarcely one of them visible at night.

use of the bi-chloride of mercury with happy effect. I recollect well its first introduction about thirty-five years ago into the Practical-Room of my excellent preceptor and valued friend Mr. Carpue, where it was highly prized by the pupils. This process, however, had also its defects. The textures were undoubtedly preserved ; but they speedily lost colour, and although the invasions of the maggots were prevented, and the atmosphere rendered more pure, the solution being used unnecessarily strong, the edge of the scalpel became speedily blunted, and the whetstone and strop were never out of the dissector's hand.

For a great many years I have in my occasional pathological examinations used a combination of the nitrate and bi-chloride, with most satisfactory results. I use from five to ten grains of the bi-chloride dissolved in a pint of water, and to this solution I add as much nitrate as it will dissolve. While the nitrate preserves the colour, the quantity of the bi-chloride is sufficient to prevent the incursions of the maggots, and the process of decomposition is thus effectually retarded for such a length of time as completely to answer every requisite purpose. Where this combination then has not been tried, I have no hesitation in recommending it as equal, if not superior, to any of those in present use.

In the preservation of timber and other perishable substances, the antisceptic property of corrosive sublimate under Kyem's patent, has now for a good many years been abundantly tested, and the subject of much notoriety : it has only failed where it has not sufficiently penetrated the substances subjected to its influence. In all my trials of its efficacy, nothing could be more satisfactory. To the naturalist I hold it to be invaluable ; for if the skin of any animal is dipped into a solution of the above mentioned strength, and thereafter dried, neither moth nor maggot will ever make any impression on it, nor will the colour of either hair or feathers be in the slightest degree injured or altered.—*London and Edin. Monthly Journal.*

